# Early postural dynamics reveal the rapid processing of fake social cues for deceptive actions in sports

**DOI:** 10.1038/s41598-026-61922-8

**Published:** 2026-07-21

**Authors:** Iris Güldenpenning, Lars Schulze Frielinghaus, Nils T. Böer, Thomas Schmidt, Matthias Weigelt

**Affiliations:** 1 Psychology and Human Movement Group, Department of Exercise & Health, University of Paderborn, Warburger Str. 100, 33098 Paderborn, Germany; 2 Exercise Science and Neuroscience Unit, Department of Exercise & Health, University of Paderborn, Warburger Str. 100, 33098 Paderborn, Germany; 3 Visual Attention & Awareness Laboratory, University of Kaiserslautern-Landau (RPTU), Kaiserslautern, Germany

**Keywords:** Neuroscience, Psychology, Psychology

## Abstract

Deceptive actions such as head fakes are a hallmark of interactive sports, yet it is not well understood how decisions and whole-body responses to head fakes unfold over time. In the present study, participants viewed video sequences of a basketball player passing to the left or right, either with or without a head fake. In the buzzer task, participants moved laterally to press a response button as if to intercept the pass. In the vocal task, they verbally indicated pass direction. Reaction times (RTs), movement times (MTs), and medio-lateral center-of-pressure (CoP) displacements were recorded to investigate whether motor output is rapid and stimulus-driven or rather requires continuous integration of fake and pass cues. Further, we investigated whether the mere observation of a basketball player leads to overt micromovements even if no response movement is intended. The data analysis shows that head fakes increased RTs in both the buzzer and vocal tasks. In the buzzer task, head fakes induced an initial, small erroneous CoP shift, which was then rapidly corrected. When CoP trajectories were aligned by head orientation, fake and non-fake trajectories diverged shortly after movement onset. In the present dynamic sport-related context, this pattern is more compatible with the early integration of head and pass information than with a strictly sequential prime-target account, although the paradigm differs from classic Rapid Chase Theory designs with discrete primes and target events. In the vocal task, participants were not instructed to move, yet CoP traces showed tiny but systematically lateralized micromovements with a temporal structure resembling that in the buzzer task, albeit without an initial erroneous shift for head fakes. These postural fluctuations are consistent with action-observation-related motor activation, although they do not uniquely identify motor resonance and may also reflect attentional or generalized embodied response tendencies. A clear deceptive response conflict at the postural level emerged only when a lateralized action was prepared.

## Introduction

In the context of interactive sports, athletes frequently employ deceptive maneuvers to enhance their performance^1^. A classic example of this is the head fake in basketball, where a player initiates a pass in one direction (e.g., left) while orienting the head oppositely (e.g., right). This mismatch has been shown to induce slower, more error-prone responses compared with genuine passes and has been termed the head-fake effect^2–5^. The head-fake effect can be explained by the Theory of Event Coding (TEC)^6^, which assumes that perceptual features and action features are temporarily integrated into “event files.” An event file binds stimulus features (e.g., pass direction, head orientation) to response features (e.g., a left vs. right response). In a genuine pass to the left (pass-left, head-left), both stimulus features converge and retrieve the same event file, which already contains the left response, thereby facilitating fast responding. In a head-fake situation (pass-left, head-right), the head feature tends to retrieve an event file associated with a right response, while the task-relevant pass direction retrieves the left-response event file. This partial retrieval of competing event files creates a competitive dynamic that must be resolved before responding, and thus, delaying responses.

The head-fake situation in a real one-on-one situation in basketball is characterized by two specific aspects: constantly changing visual input due to the attacker`s action and the requirement for a complex response movement by the defender. These two aspects distinguish the head fake situation from most standard laboratory settings. The present study therefore investigates a component of TEC that might be specific to such realistic situations, namely the process by which dynamic visual input is handled when competing event files are retrieved. Two approaches seem to make plausible predictions for the basketball setting: the Continuous Flow Model^7^ and Rapid-Chase Theory^8,9^. Both accounts are explained below. Beyond that, TEC assumes a close functional coupling of perception and action, which prepares or facilitates the execution of an action. The extent to which this preparation remains ‘below the threshold’ (i.e., only as internal simulation, covert activation) or manifests itself as a measurable (micro-)movement (i.e., minimal shifts of the body`s Center of Pressure [CoP]) is not specified within TEC. The present study therefore investigates whether the mere observation of a basketball player performing a head fake leads to overt (micro-)movements and a detectable response conflict.

A model that seems suitable for representing the visuo-motor requirements of dynamic real-world situations is the Continuous Flow Model^7^. A salient assertion of this model is that, with the onset of visual input, multiple channels simultaneously process features of the visual input, thereby activating a broad spectrum of responses that are nevertheless suppressed. As the process continues, the accumulation of visual information leads to the narrowing of the activation of responses to select few suitable candidates until one exceeds the evocation threshold. Subsequent to the removal of its inhibition, the response is initiated. This approach attributes greater flexibility in action, as they can be adapted to changing visual input^10^.

Continuous information flow can be demonstrated in interference tasks for finger and mouse movements toward the corners of a monitor^11–13^. In incongruent conditions, the task-irrelevant feature, or cue, would require a pointing movement to the wrong corner of the monitor. General findings for the Simon-task, the Eriksen-flanker task, and the Stroop task are that interference can become visible both in RTs and in MTs^10,14^. In incongruent conditions, longer MTs are observed as the initial movement is initiated in the wrong direction and requires correction. This can be seen, among other dependent variables (e.g., area under the curve; AUC), from the recorded movement paths^14^. Deviating findings from those referred to above probably depend on the respective experimental setting (e.g., time limit for response initiation; response device, cf.)^14^.

An important characteristic of head fakes in basketball is that the deceptive head turn occurs prior to the pass^15^. This temporal sequence suggests a priming-like architecture: early head-orientation information could activate a corresponding response tendency before pass-direction information becomes available. Rapid-Chase Theory (RCT) provides a strong version of such a priming account^8,9^. It assumes that prime and target are controlling the motor output in strict temporal sequence: early response activation is time-locked to the prime, whereas later target information can take over and redirect the response. A key diagnostic prediction is the *independence criterion*: initial motor output should depend exclusively on the prime and thus, be invariant with respect to all target characteristics. In contrast, if prime and target were processed concurrently and not in strict sequence, the earliest motor output should depend jointly on both prime and target characteristics, not on prime characteristics alone.

Converging evidence for RCT comes from studies by Schmidt and colleagues using speeded pointing responses in which a brief prime preceded the target and prime-target stimulus-onset asynchrony (SOA) was systematically varied^8,9,16,17^. Continuous trajectories showed that initial responses exclusively followed the prime: congruent trials proceeded directly towards the goal, whereas incongruent trials produced initial detours toward the prime or delayed movement onsets. Crucially, the earliest movement segment was invariant across target characteristics, and thus, meeting the independence criterion. Importantly, this prime-driven initial invariance has been observed across different stimulus materials (for red and green color stimuli^8^, for natural images of animals vs. non-animals, the discrimination of simple geometric objects [both^17^]), and for different motor output (e.g., continuous trajectories^16,17,18^, isometric response force^19^ and electrophysiological markers^20^), suggesting that rapid-chase dynamics can generalize across response systems.However, evidence for RCT in complex whole-body movements, such as defensive movements in basketball, is still lacking. Importantly, unlike classic RCT paradigms that systematically vary prime-target SOA with discrete prime/target events, the head fake is dynamic and overlapping with the subsequent pass. It is therefore an open question whether RCT can explain the head-fake situation at all.

A plethora of studies have previously investigated the head-fake effect in basketball^2,3,21^, however, only one study evaluated CoP trajectories^22^. Schütz et al.^22^ investigated whether the head orientation of a non-consciously perceivable basketball player (i.e., a masked prime) could affect a complex motor response (i.e., body movement to the left/right as if to intercept the pass). During the experiment, participants stood at a force plate, and the mediolateral displacements (i.e., to the left/right side) of the CoP were measured. The study demonstrated that for congruent prime-target arrangements (i.e., the head orientation in the prime picture was congruent to the pass direction in the target picture), participants initially shifted the CoP in the opposite direction, that is, to the contralateral leg, and then pushed off in the response direction. For incongruent prime-target arrangements (i.e., the head orientation in the prime picture was incongruent to the pass direction in the target picture), the initial shift of the CoP was to the ipsilateral leg, followed by the response action previously described. Consequently, in case of an incongruent prime, the body was initially shifted to the incorrect side. The erroneous shift was induced by a preceding subliminal presentation of the image of a basketball player with his head turned to one side.

**Fig. 1:**
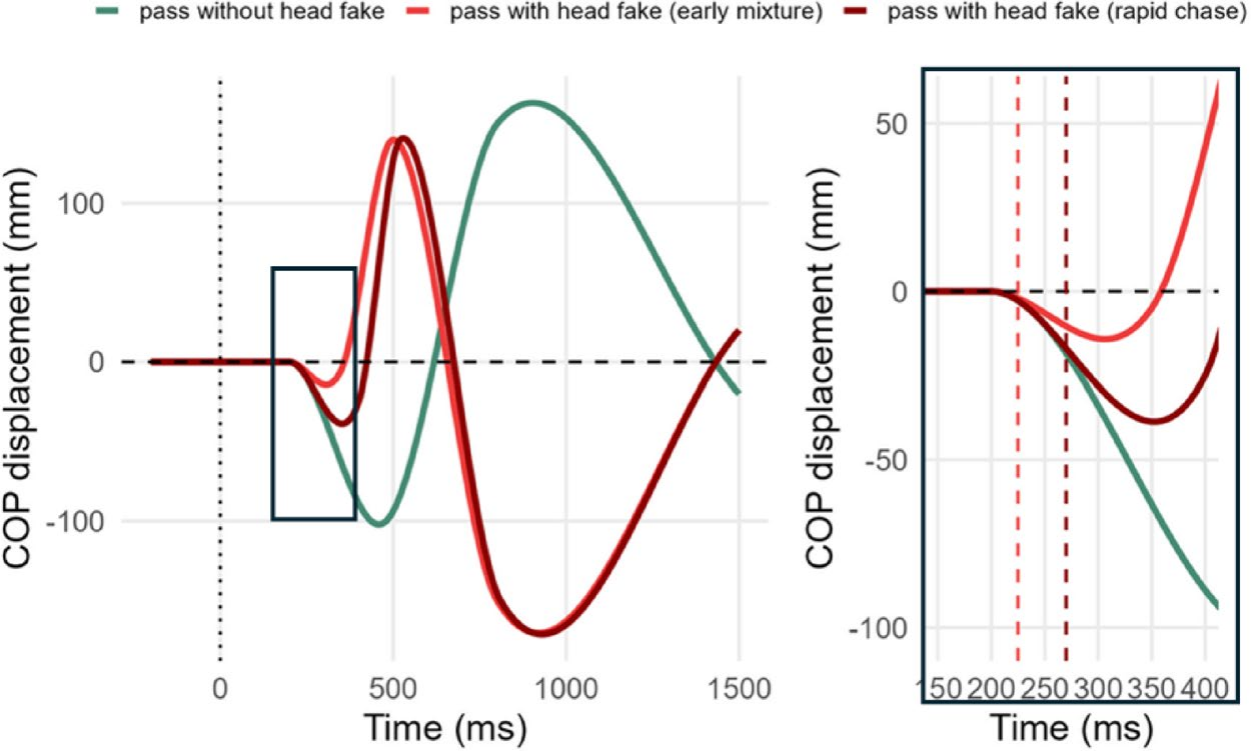
Expected CoP displacement for different visuo-motor processes. *Note*. The image on the left shows how a head fake would affect the initial erroneous movement in dependence of the visuomotor process (i.e., rapid chase vs. early mixture). The green line depicts movement shifts to passes without head fakes and the red/orange lines depict movement shifts to passes with head fakes. To compare the processing of the head orientation in dependence of further information, the depicted CoP shifts are aligned regarding head orientation (i.e., same head orientation, different pass direction). The image on the right zooms in on the relevant area: the dashed vertical lines illustrate the branch-off times between the pass without head fake and the pass with head fake. RCT would predict that the initial CoP shift is identical for passes with head fakes and for passes without head fakes and the branch-off between the pass without head fake and the pass with head fake would occur after some time of identical course (red line). In contrast, continuous flow theory predicts an early mixture of head fake and basketball pass information to control even the earliest responses. As a result, there would be no initial invariance, and the orange and green curves would diverge right from the start of the movement.

Schütz et al.^22^ also investigated whether the intention to move to either side is the prerequisite for unconsciously induced movement shifts. They therefore also measured CoP displacements when the participants responded with simple button presses (instead of body movements) while standing on the force plate. In this setting, no CoP displacements occurred. The authors concluded that the intention to move to either side is essential for unconscious behavioral priming. But what happens when social stimuli (e.g., head turn) are consciously presented, but there is no intention to act? This question is at the heart of the second objective of our study. It is generally accepted that the observation of actions activates the corresponding action representation in the observer^23^, which has been termed motor resonance^24^. This action system facilitates the understanding of another’s intention^25^, the anticipation of their action^26^, and is paramount for the coordination of collaborative actions among individuals^27^. Furthermore, in social contexts, the observation of actions frequently results in the imitation of the observed behavior^28^. However, it should be noted that not all observations of actions necessarily result in observable tendencies to act, despite the activation system in the brain. Therefore, the present study sought to ascertain whether observing head fakes would result in overt (erroneous) movements even when no action is intended (Fig. 1).

### The present study

**Fig. 2:**
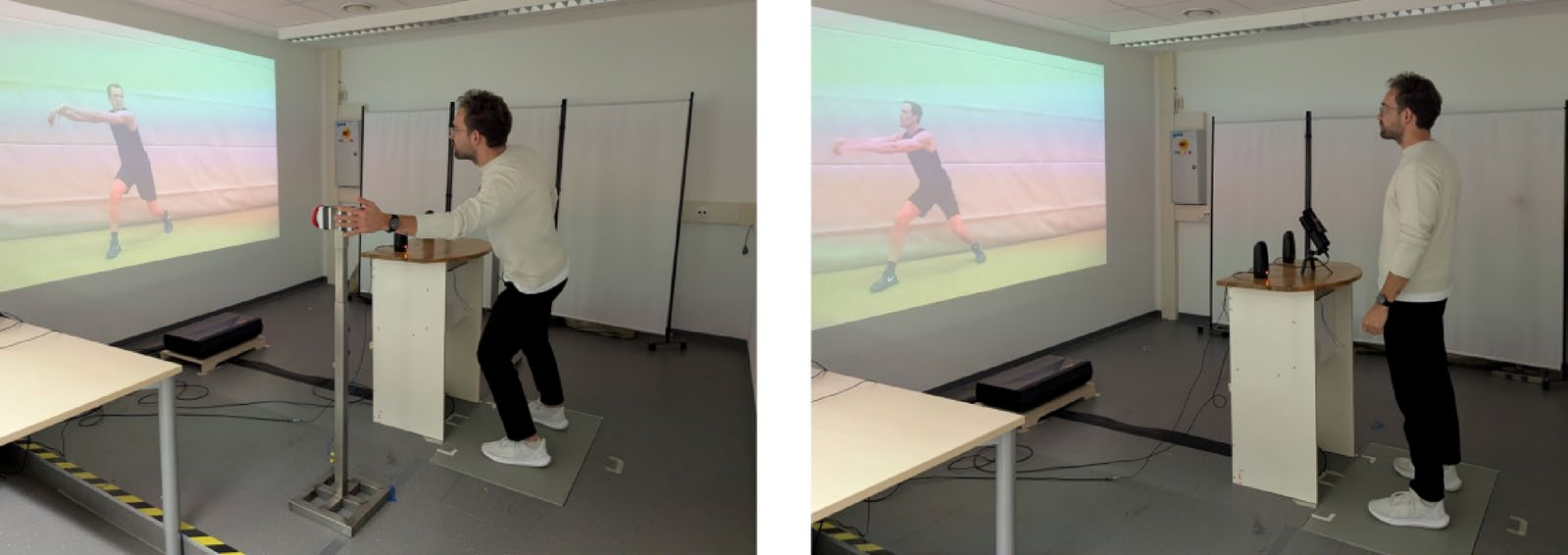
Experimental set-up of the study. *Note.* The left picture represents the set up for the buzzer task. The right picture represents the set up for the vocal task. Written informed consent for publication of these images in an online open-access journal was obtained from the person shown in the photograph.

Two variants of the head-fake task were conducted in the present study, the *buzzer task* for working on the first aim (i.e., visuo-motor processing of head fakes) and the *vocal task* for the second aim (i.e., automatic activation of movement tendencies). In both tasks, participants were asked to react as fast and accurately as possible to video sequences of a basketball player. In the buzzer task, the required response was a whole-body movement to one side as if to intercept the perceived pass (cf. Fig. 2). In the vocal task, participants were asked to verbally indicate the pass direction of the observed player. In addition to RTs and MTs (only for the buzzer task), medio-lateral displacements of the CoP were measured.

*Buzzer Task.* According to TEC^6^, divergent spatial stimulus codes for pass direction and head orientation (i.e., left, right) activate competing event files. In accordance with previous studies on the head-fake effect in a realistic-like setting (e.g.,)^3^, we therefore expect a head-fake effect in RTs and MTs. With respect to CoP displacements for reactions to a pass without a head fake, an initial shift to the contralateral side (i.e., to the right) is expected, to push off with this leg towards the response button on the ipsilateral side (left) (cf.,)^22^. For reactions to a pass with a head fake, we expect an initial erroneous shift towards the ipsilateral (i.e., left) response button. Of particular interest was whether the timing and progression of the CoP shifts would show a pattern resembling a strict prime-dominated sequence, as proposed in Rapid-Chase Theory, or a pattern more consistent with early mixture or continuous integration of available visual information. Because the present stimuli were dynamic videos rather than discrete prime-target displays, this comparison should be understood as an extension of these theoretical ideas to a more naturalistic sports-related context rather than as a direct test of RCT in its classic form. Indicative for RCT would be the *independence criterion*. Because RCT postulates that visuomotor processing of the prime and target occurs in strict sequence, it predicts that this strict sequence is also observed in the motor output. The very first phase of the overt response should therefore only depend on characteristics of the prime (head fake), but not on any characteristics of the target (pass direction). This is illustrated in Fig. 1, where the initial time-course to the pass with head fake (red line) is initially identical to the pass without head fake (green line) and only branches off from the common time-course later in processing when target information starts to influence the response. In contrast, if processing of the two stimuli were not strictly sequential, we would expect the initial motor output to be affected not only by the head fake, but also by pass direction. In such an “early mixture” model (orange line), the earliest overt motor output would depart from the green curve shortly after movement onset of the overt response, and no initial invariance of the trajectory would be observed. Initial invariance in pointing trajectories has been observed in response priming tasks with short SOAs and fast responses to the target^17^, whereas early mixture has been observed in tasks with long SOAs that forced participants to keep prime and target in working memory before initiating the response^29^.

*Vocal task.* It is hypothesized that the spatial stimulus codes of the pass direction and the head orientation (i.e., left, right) activate corresponding vocal response codes (left/right) in a manner analogous to that of a body response movement. Consequently, a head-fake effect occurs in RTs. Furthermore, as the response cannot be corrected after speech onset, some errors will inevitably occur. Head-fake stimuli will produce more errors than stimuli without head fakes. With respect to the displacements of the CoP, the stimuli of the basketball player, who performs body movements to the left/right side, might lead to an internal activation of these actions in specific brain areas of the observer (i.e., premotor cortex, posterior/parietal cortex). This internal activation could result in small displacements of the CoP, similar to those explained for the buzzer task. Slight postural shifts to the left/right are expected to be consistent with action-observation-related motor activation, such as motor resonance^23^, although such shifts could also reflect attentional orienting or more general embodied spatial-response tendencies.

## Methods

### Participants

Planning of the sample size was carried out using MorePower 6.0.4^30^. Previous studies, which investigated the head-fake effect with video stimulus material^31–33^, reached very large effect sizes (ɳ_p_^2^ > 0.90). A large effect size (ɳ_p_^2^ = 0.40), with power set at 0.95, for a repeated measures design with the factor *type of pass* (pass with head fake vs. pass without head fake), yielded a recommended total sample size of 22.

Initially, 28 participants were tested. Eight participants had to be excluded because of technical problems unrelated to participant performance or experimental condition. Specifically, participants 1–5 were excluded because of force-plate recording problems during the initial setup phase, and participants 21, 22, and 24 were excluded because of synchronization problems between the force-plate data and the behavioral response data. These exclusions were made prior to the inferential analyses and were entirely technical in nature. The final sample therefore consisted of 20 participants (9 females, 11 males; mean age = 22.4 years, *SD* = 1.3). The final sample was slightly smaller than the sample size of 22 participants, which was targeted a priori. Thus, although the study retained sufficient sensitivity for large effects, such as the expected head-fake effect in RTs, the reduced sample size should be considered when interpreting smaller effects, especially in the dynamic CoP measures and error-rate analyses. All participants were sport science students from the University of Paderborn without specific experience in basketball. All participants volunteered and provided written informed consent. All rights of the participants were protected, and the study was carried out according to the sixth revision (Seoul) of the 1964 Declaration of Helsinki by the World Medical Association. This research was also reviewed and classified as ethically inoffensive by the Ethics Committee of the University of Paderborn. No identifying information was obtained from the participants, apart from their age and handedness. The present article does not include any identifying or potentially identifying information to which participants did not consent.

### Apparatus and stimuli

A short-distance laser projector (Optoma CinemaX D2 HDMI RJ-45) was used to present the stimulus material on a white wall of the laboratory. Participants were positioned at the force plate (AMTI-BP600900, 60 cm x 90 cm), 3 m away from the wall. Participants adopted a feet parallel position, which was standardized by using a marker in the center of the force plate as a reference point. The AMTI force plate was connected with a 64-channel A/D converter (16-bit resolution, 1000 Hz sampling frequency). Force plate data (i.e., ground reaction forces and torques) were recorded using Simi Motion (Simi Reality Motion Systems, version 9.0.2).

For the vocal task, a microphone (RØDE NT-USB) was placed on a small table in front of the participants (cf. Fig. 2). The microphone was connected via a USB-port. For the buzzer task of the experiment, two start buttons were installed on a small table at a height of 100 cm in front of the participants (cf. Fig. 2). The response buttons were located on a movable custom-made steel-system at a height of 120 cm to the right and left of the participants. The distance between the response buttons was adjusted to the height of the participants (span + 15 cm). All buttons of the setup were soldered to the buttons of a custom mouse and connected with the stimulus presentation and data recording computer via a USB-port.

**Fig. 3:**
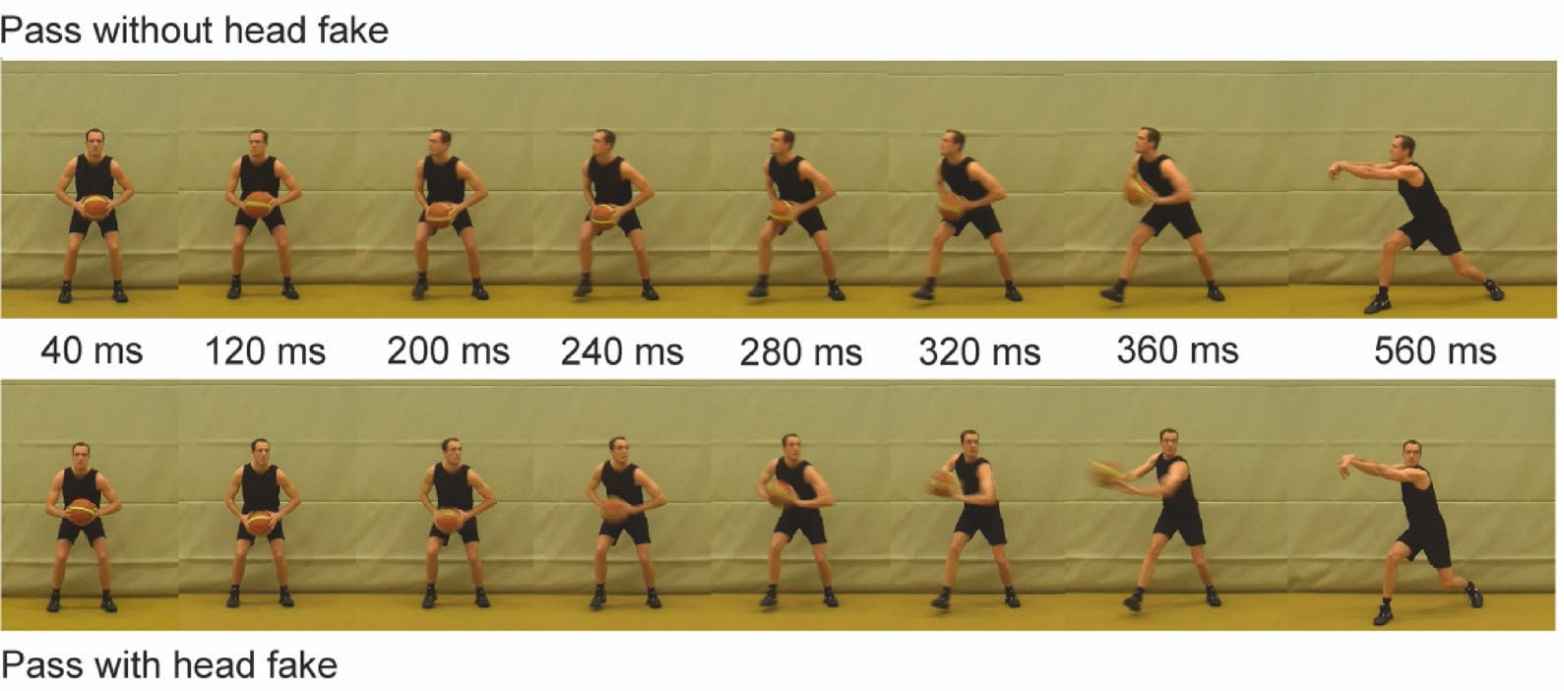
Stimulus material used in the study. *Note.* Video sequences of a pass without a head fake (upper panel) and a pass with head fake (lower panel), depicted as a series of “frozen” picture frames of that sequence. Written informed consent for publication of these images in an online open-access journal was obtained from the person shown in the photograph. Used with permission of Acta Psychologica, Human Movement Science, and International Journal of Sport and Exercise Psychology.

Video sequences of a male basketball player wearing a black shirt and black shorts were used as stimulus material (Fig. 3). During the video recording, the basketball player stood in front of a gray wall. Before each pass, the basketball player held the ball in his hands, centered the ball in front of his body, and looked directly at the camera. It was recorded how the basketball player passed the ball from this starting position to the left or right, looking in the same direction (pass without head fake) or in the opposite direction (pass with head fake). For the experiment, only video sequences with comparable spatio-temporal parameters (duration, movement of the model, movement of the ball) were used. That is, the duration from the initiation of the throw (initial left/right movement of the ball) to the end of the throw (ball leaves the hands) was the same for passes with and for passes without head fakes.

The videos were presented at 25 frames per second; thus, each displayed frame corresponded to 40 ms. For the frame-based characterization of the video material, the initial frame at stimulus onset was labeled frame 0 and was displayed from 0 to 40 ms. Frame 1 was displayed from 40 to 80 ms, frame 2 from 80 to 120 ms, and so forth. Thus, when an event is described as occurring in a given frame, this refers to the frame in which the respective event was first visible. The last displayed frame was frame 18; consequently, each video consisted of 19 displayed frames including frame 0, resulting in a total duration of 760 ms. A frame-based characterization was conducted to describe the temporal structure of the fake and non-fake videos, which showed that the fake and non-fake videos matched with respect to several central action events. At the beginning, the video sequences showed the basketball player standing still in the initial position (0–80 ms). Afterwards, the basketball player initiated the head rotation and started to shift his body mass into the pass direction (160 ms), before the ball left the players’ hands (360–400 ms). The passing movement ends with fully stretched arms (560 ms). The video finished after 760 ms. Notably, for passes without head fakes, the onset of head movement was first visible in frame 3 (120 ms). In the head-fake videos the head rotation started between frame 3 and frame 4, that is, around the transition from 120 to 160 ms.

Two video sequences, one pass without and one pass with head fake, were mirrored along the vertical axis. The resulting four video sequences formed the stimuli for the experiment. Videos were presented with a size of 275 cm × 150 cm.

The presentation of the stimulus material and data recording was controlled using an IBM-compatible personal computer. For synchronization purposes, serial port sequences were sent to the Simi Motion output files at the moment of target presentation.

### Procedure

The whole study lasted about 40–45 min. Before participants were placed at the force plate, they received written instructions about the procedure of the study and the two different tasks. In the buzzer task, participants were asked to react as fast and as accurately as possible to the direction of the pass by pressing the left or right response button as if they were trying to intercept the perceived pass. In the vocal task, the direction of the pass had to be named verbally (i.e., left/right) as fast and as accurately as possible. The order of the two tasks was counterbalanced across participants. There were four different trial conditions resulting from the 2 × 2 combination of the factors head turn (left, right) x pass direction (left, right): passes without head fakes to the left or right side and passes with head fakes to the left or right side.

Apart from the answer, the procedure was identical in both tasks: Each trial started with the presentation of a black screen (500 ms), followed by a black screen with a white fixation cross in the center (500 ms), and the target (until response). Incorrect responses elicited visual error feedback (“Fehler”; German word for “error”). In the buzzer task, participants had to replace both hands at the start buttons to initiate the next trial. Each experimental version consisted of five blocks with 40 randomized trials. Participants performed sixteen practice trials before each task.

### Data analysis applied to both tasks

#### Pre-processing

In the buzzer task, trials were excluded if (1) RT < 100 ms or RT > 1000 ms and (2) MT < 100 ms or MT > 800 ms (1.3% of trials). In the vocal task, trials were excluded if (1) RT < 100 ms or RT > 1000 ms and (2) the verbal response was incorrect (3.1% of trials).

#### Discrete performance measures (RT, MT, ER)

For the buzzer task, the effect of *type of pass* (pass with head fake vs. pass without head fake) was analyzed for reaction time (RT) and movement time (MT). RT was defined as the time from target video onset until lifting the hands off the start buttons. MT was defined as the time from lifting the hands off the start buttons to pressing the response buzzer.

For the vocal task, the effect of *type of pass* was analyzed for RT, defined as the time from stimulus onset to speech onset. Error rates (ER) were computed for both tasks. No incorrect responses occurred in the buzzer task; therefore, ER was not analyzed further in this task. In the vocal task, ER was analyzed as a function of *type of pass*.

#### Force-plate processing and CoP computation

Force-plate data were imported into MATLAB (v.2023b, Mathworks Inc., USA) and smoothed using a 10 Hz, 4th-order, zero-lag Butterworth low-pass filter. Medio-lateral CoP displacement was computed as My/Fz. Using the synchronization signal, the continuous data stream was segmented into 1700-ms epochs (− 200 to + 1500 ms relative to stimulus onset). For each participant and condition, CoP trajectories were averaged across trials. To account for small baseline offsets at stimulus onset, the CoP time series was baseline-corrected by subtracting the CoP value at stimulus onset from the entire epoch. Further analyses were conducted in R. The R-scripts were created with the assistance of ChatGPT-5.2 Thinking and are available in the OSF for this study.

#### Alignment of conditions

For CoP analyses, the conditions were aggregated for responses to the left and right within each level of *type of pass*. To align trajectories for passes with and without head fakes with respect to head orientation (rather than pass direction), CoP signs were inverted for (a) leftward responses in non-fake trials and for (b) rightward responses in fake trials. Accordingly, trajectories for fake and non-fake trials were directly comparable under identical head-orientation alignment.

### CoP onset estimation and peak detection

To obtain robust group-level onset estimates, we applied a leave-one-participant-out jackknife procedure following Ulrich and Miller^34^ and related work on time-resolved motor output [e.g., 8]. For each condition, we first estimated the onset latency from the full-sample grand-average trajectory (i.e., the mean trajectory including all participants). Onset latencies were defined using a sustained threshold-crossing criterion (“first sustained threshold crossing”). The baseline interval was defined as the 200 ms before stimulus onset. For each grand-average trajectory, we computed the standard deviation of the baseline interval and defined an effective amplitude threshold *T*_eff_ = max(*k* * *SD*_baseline_, *T*_min_ ). The effective amplitude threshold ${T}_{eff}$ represents the minimum CoP displacement that must be exceeded to be considered a meaningful, reliable deviation from baseline, because it takes the more conservative (larger) of (a) a noise-based threshold derived from baseline variability ($k\cdot S{D}_{baseline}$) and (b) a task-specific minimum absolute displacement ${T}_{\mathrm{m}\mathrm{i}\mathrm{n}}$. In the buzzer task, we used *k* = 4 and *T*_min_ = 0.7 mm; in the vocal task, we used *k* = 2 and *T*_min_ = 0.05 mm, reflecting the smaller displacement magnitude. Onset was defined as the first time point *t* at which $\mid x(t)\mid >{T}_{eff}$ for at least $m=30$ consecutive samples. Because the force plate was sampled at 1000 Hz, this criterion corresponds to a sustained deviation of at least 30 ms. This requirement was included to reduce the likelihood that brief fluctuations around baseline were misclassified as movement onsets. The task-specific minimum displacement criteria were chosen because the magnitude of CoP displacements differed markedly between tasks: the buzzer task involved pronounced whole-body movements, whereas the vocal task involved only small postural micromovements. Diagnostic plots were used to verify that the selected parameters produced plausible onset estimates and did not identify isolated baseline fluctuations as movement onset. These diagnostic plots and analysis scripts are available on the OSF repository (see open practice statement).

To estimate uncertainty of the onset latencies, we computed jackknife subsample estimates by repeatedly omitting one participant and re-estimating onset latency from the grand-average trajectory of the remaining participants. For the fake-non-fake comparison, leave-one-out difference scores were computed and used to estimate the jackknife-based standard error of the full-sample onset difference^34^. This approach preserves the group-level nature of the onset estimates while providing an uncertainty estimate based on the jackknife subsamples.

For descriptive characterization of CoP dynamics, peak latencies and corresponding peak values were extracted from the full-sample grand-average trajectories. Peaks were defined as local extrema after movement onset. No inferential statistics were conducted for peak measures.

### Data analysis specifically applied to the buzzer task

#### Slope analysis (early dynamics)

The slope analysis was designed to characterize the earliest post-onset dynamics of the CoP trajectories in the buzzer task. The analysis started at 200 ms after stimulus onset because the jackknife onset analysis estimated the onset of the initial CoP response at 212 ms for non-fake trials and 218 ms for fake trials (see below). Thus, the first analysis window, 200–225 ms, covered the earliest time range around the estimated onset of postural response initiation. We analyzed four consecutive 25-ms windows from 200 to 300 ms. This interval was chosen because it captures the early post-onset phase of the CoP response and precedes the first local extremum of the erroneous initial shift in head-fake trials. The 25-ms window size was selected to provide a local estimate of trajectory direction and rate of change while preserving sufficient temporal resolution to detect rapidly emerging condition differences.

Within each time window, CoP values were regressed on time (ms) using ordinary least squares separately for each participant and condition. The regression coefficient was taken as the slope and converted to mm/s for reporting. Condition differences between non-fake and fake trials were tested against zero using paired-samples* t*-tests for each time window, with Holm correction across the four tests. The onset-latency analysis and slope analysis addressed complementary questions: the onset analysis tested whether the first detectable CoP response began at different times between conditions, whereas the slope analysis tested whether the early post-onset trajectory dynamics differed once the response had begun. To examine whether the pattern depended on the specific 25-ms windowing choice, we additionally conducted exploratory robustness checks using smaller 10 ms and 20 ms windows and with taller 50-ms windows and with 25-ms windows shifted by 10 ms. All of these analyses yielded the same pattern of early condition differences and are also provided in OSF repository (see open practice statement).

## Results

### Discrete performance measures (RT, MT, ER)

**Fig. 4:**
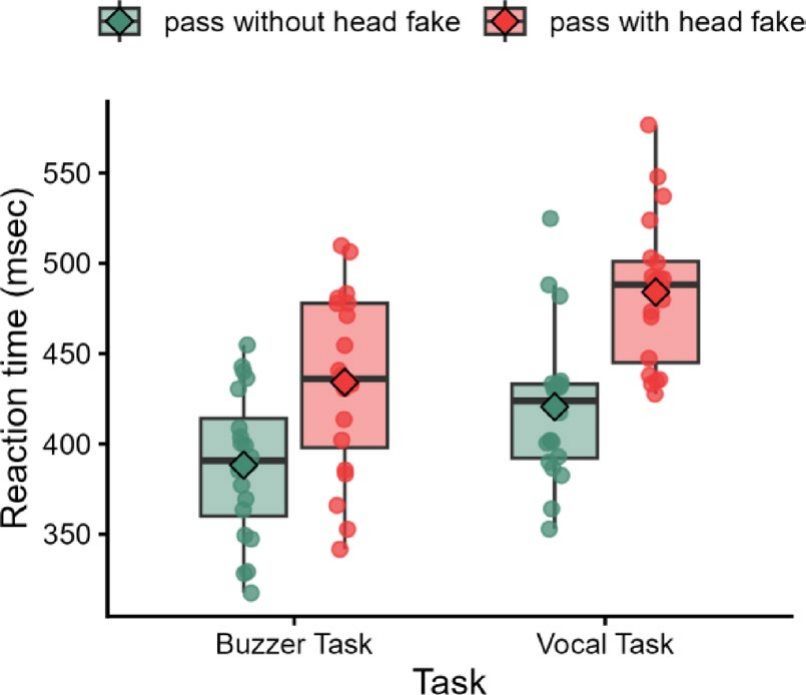
RTs for passes with head fakes and for passes without head fakes for the buzzer task and the vocal task. *Note*. Reaction times (ms) in the buzzer task and the vocal task as a function of type of pass (pass without head fake vs. pass with head fake). Boxplots ^35^ summarize the distribution of participant-level RTs; individual participant observations are overlaid as points, and the mean for each condition is indicated by a diamond.

*Buzzer task.* RT data are visualized in Fig. 4. Analyses of the RT data showed that participants reacted faster to passes without head fakes (*M* = 388 ms; 95% CI = [369; 407]) than to passes with head fakes (*M* = 434 ms; 95% CI = [411; 458]). A *t*-test for paired samples revealed a significant head-fake effect (*M* = 46 ms; 95% CI_Diff_ = [39; 53]), *t*(19) = 13.027, *p* < 0.001, *d* = 2.91. Analyses of the MTs depicted a comparable pattern, that is, faster movement times for passes without head fakes (*M* = 318 ms; 95% CI = [281; 355]) than for passes with head fakes (*M* = 341 ms; 95% CI = [312; 370]). This difference in movement times (*M* = 23 ms; 95% CI_Diff_ = [4; 43]) was qualified by a *t*-test for paired samples, *t*(19) = 2.576, *p* = 0.019, *d* = 0.58.

*Vocal task.* RT data are visualized in Fig. 4. Analysis of the RT data showed that participants responded faster to passes without head fakes (*M* = 421 ms; 95% CI = [401; 440]) than to passes with head fakes (*M* = 484 ms; 95% CI = [465; 503]). A *t*-test for paired samples revealed a significant head-fake effect (*M* = 64 ms; 95% CI_Diff_ = [54; 73]), *t*(19) = 14.047, *p* < 0.001, *d* = 3.14. Participants also produced descriptively more errors in response to passes with head fakes (*M* = 1.3%; 95% CI_Diff_ = [0.3; 2.3]) than in response to passes without head fakes (*M* = 0.7%; 95% CI = [0.2; 1.2]), however, this difference did not reach significance (*p* = 0.258).

### Dynamic measures

#### Buzzer task

**Fig. 5:**
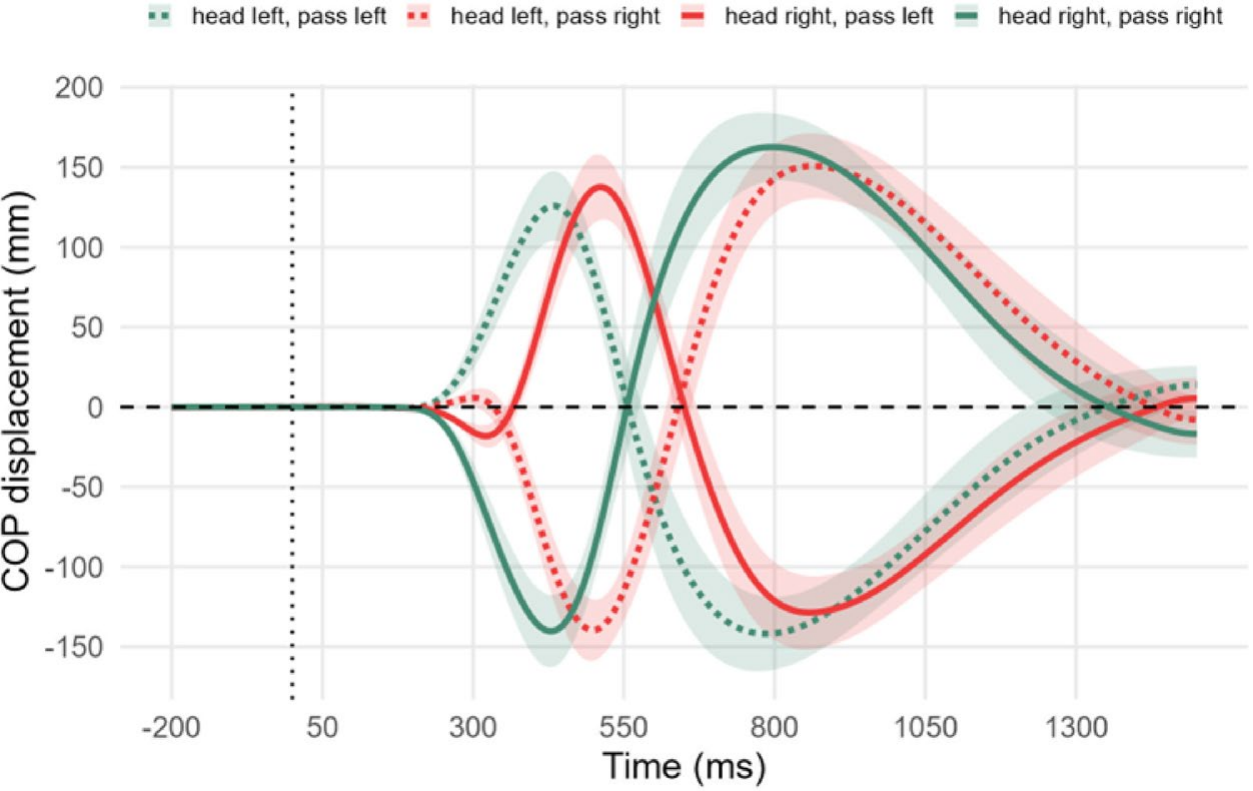
CoP-displacement for the buzzer task. *Note.* Red lines depict passes with head fakes and green lines depict passes without head fakes. The 95% confidence interval is shown transparently for the respective condition.

Figure 5 shows participants’ CoP trajectories after the start of the four different variants of the video sequences (i.e., pass to the left/right without/with head fake).

*Onset latencies and peaks* . The response movement to passes without head fakes can be described as follows. Participants initially move away from the response buzzer with an onset latency of 212 ms. At 432 ms, participants reach the peak value of this shift (peak 1; -133 mm) and start to move in the direction of the response buzzer, which they press after 706 ms. Shortly afterwards, they reach the maximum displacement in the dsirection of the response buzzer at 790 ms (peak 2; 152 mm). The initial shift away from the response buzzer is a weight shift to the contralateral leg to push off with this leg in the direction of the response buzzer^22^.

The response movement to passes with head fakes can be described as follows. Participants initially move towards the response buzzer with an onset latency of 218 ms, which is only descriptively later than after a pass without a head fake (*t*(19) = 0.606, *p* = 0.552, 95% CI = [-14.7; 26.7]). At 312 ms participants reach the peak value of this shift (peak 1; -11.4 mm) and start to move in the correct direction, that is, they shift their weight to the contralateral leg to push off with this leg in the direction of the response buzzer (peak 2; 506 ms, 138 mm). They press the response buzzer after 775 ms. Shortly afterwards, they reach the maximum displacement in the direction of the response buzzer at 862 ms (peak 3; -140 mm).

**Fig. 6:**
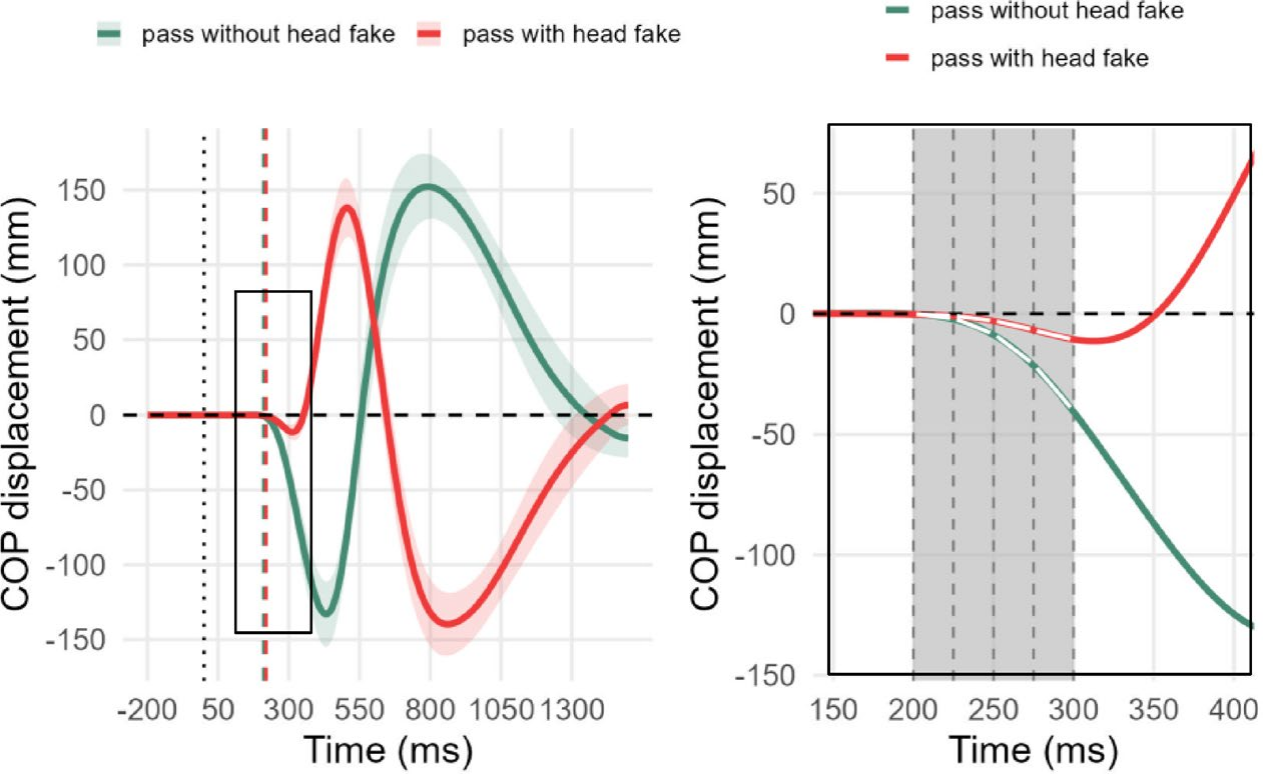
CoP-displacement in the buzzer task for both types of passes. *Note.* The folded lines depict passes with and without head fakes with identical head orientation, but with different pass direction. For passes without head fake (green line), negative shift values are movements away from the response buzzer and positive shift values reflect movements towards the response buzzer. For passes with head fake (red line), negative shift values are movements in the direction of the response buzzer and positive shift values reflect movements away from the response buzzer. The image on the right zooms in on the relevant area and depicts the four different time windows of the slope analysis. This illustration shows that the slopes for passes without head fakes and for passes with head fakes differed shortly after movement onset. In the present dynamic video paradigm, this pattern is more consistent with early integration of head and pass information than with an initially invariant, strictly prime-driven trajectory.

*Slopes*  (cf. Fig. 6, right panel). The slope difference (non-fake vs. fake) was significantly negative in every pre-defined time window, indicating a consistently steeper (more negative) rate of change in the non-fake condition compared with the fake condition throughout all periods: 200–225 ms (mean difference = -55.6, *p_*adj = 0.002), 225–250 ms (-172, *p_*adj < 0.001), 250–275 ms (-357, *p_*adj < 0.001), and 275–300 ms (-609, *p_*adj < 0.001). The magnitude of the negative difference increased monotonically across successive time windows, suggesting that condition-related divergence in CoP dynamics emerged shortly after 200 ms and became progressively stronger up to 300 ms.

### Vocal task

**Fig. 7:**
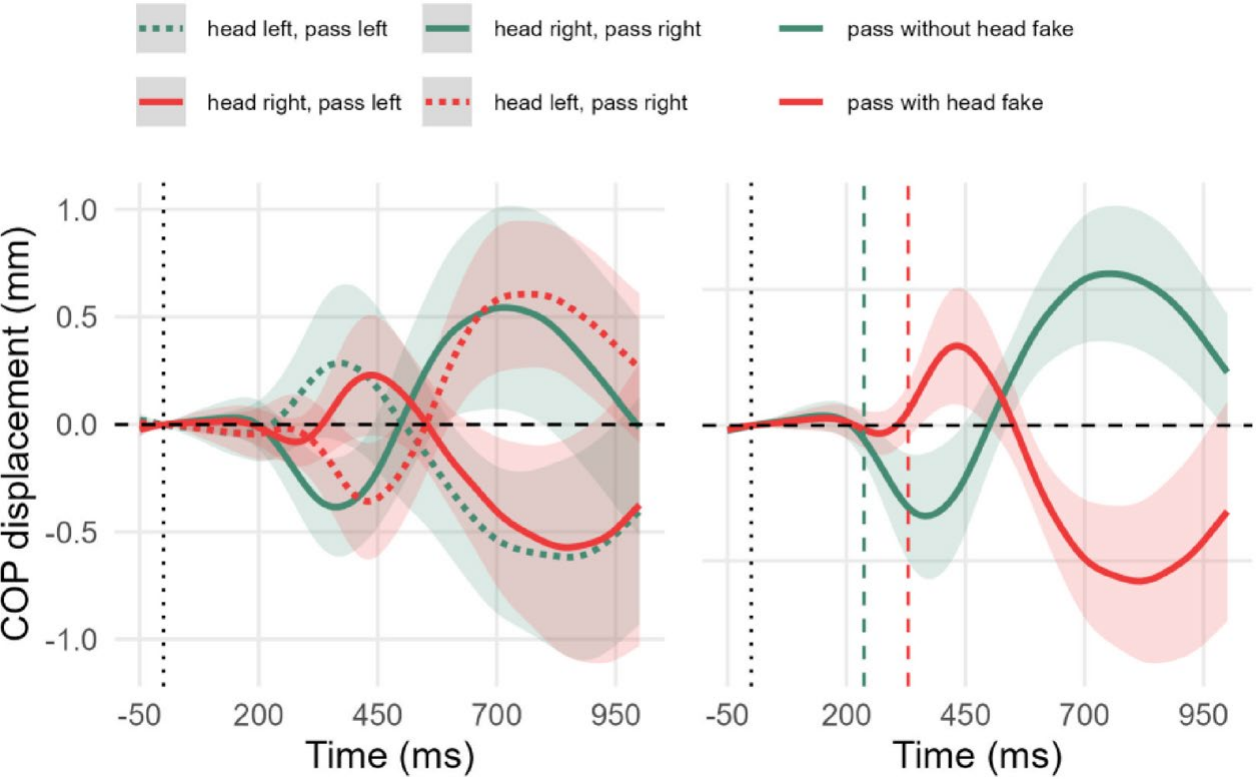
CoP-displacement in the Vocal Task. *Note.* In the left panel red lines depict passes with head fakes and green lines depict passes without head fakes. The 95% confidence interval is shown transparently for the respective conditions. In the right panel, the folded lines depict passes with and without head fakes with identical head orientation, but with different pass directions. For passes without head fakes (green line), negative shift values are movements away from the response buzzer and positive shift values reflect movements towards the response buzzer. For passes with head fake (red line), negative shift values are movements in the direction of the response buzzer and positive shift values reflect movements away from the response buzzer. The dashed vertical lines depict the initial movement onsets. Although there is no evidence that head orientation causes an erroneous shift, it shows that slight movement shifts are triggered even by the vocal task.

Figure 7 (left panel) shows participants’ CoP trajectories after the start of the four different variants of the video sequences (i.e., pass to the left/right without/with head fake). Passes to the left and right, either with or without head fakes, produced symmetrical CoP-shifts and were folded for further evaluation (Fig. 7, right panel; see section Alignment of conditions).

**Fig. 8:**
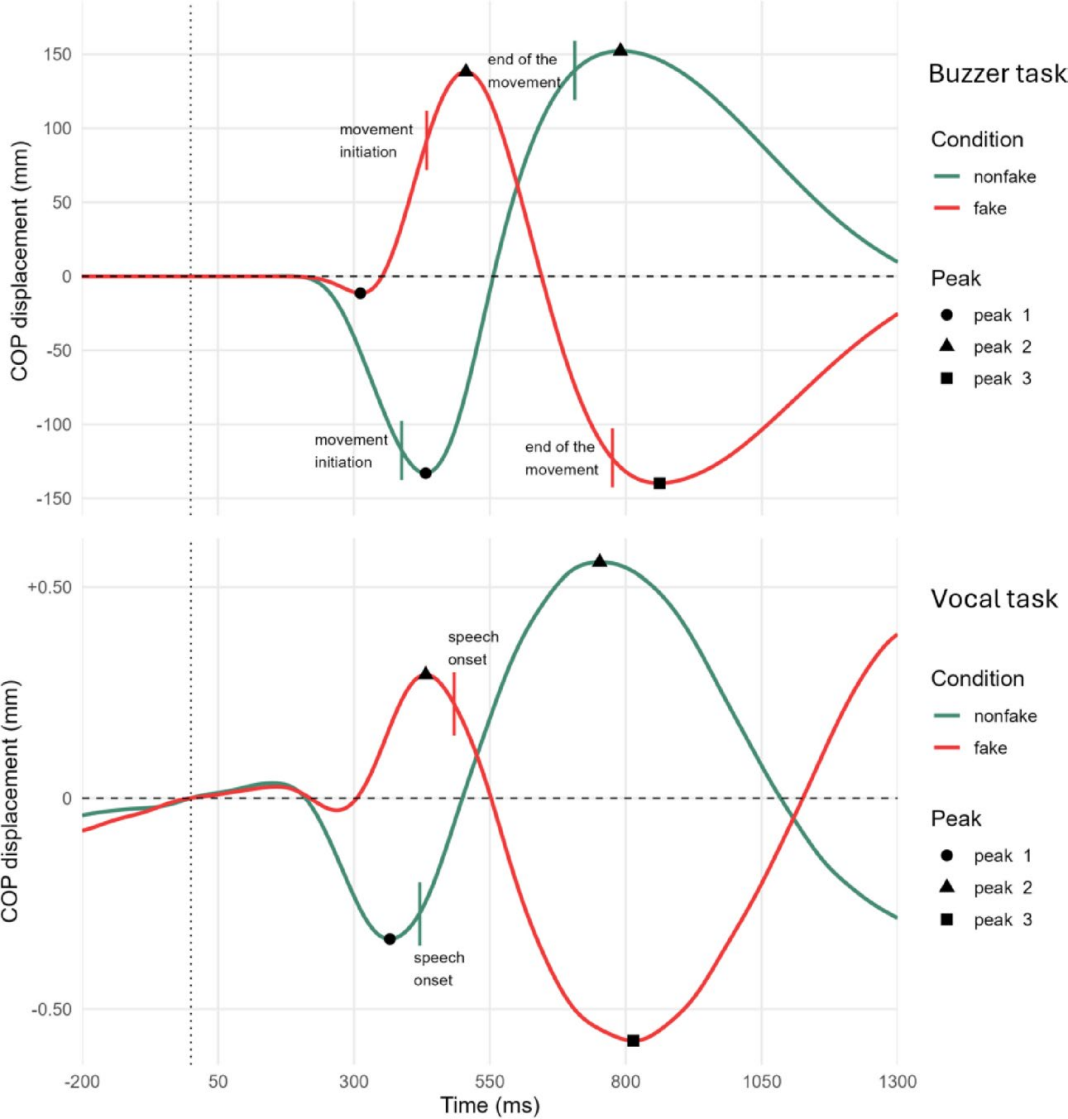
Overview of the discrete and the dynamic measurements for both tasks. *Note.* Grand-average CoP-displacement trajectories (mean across participants) are shown for the buzzer task (upper picture) and the vocal task (lower picture) to illustrate their qualitative similarity. Short vertical ticks indicate events gained from the discrete measures. Buzzer task: “Movement initiation” reflects the RT, “End of the movement” reflects the sum of RT and MT and the point in time after stimulus onset when participants pressed the response buzzer. Vocal task: “Speech onset” reflects the RT. Notably, there is no peak 1 in the vocal task for passes with head fakes, as peak detection was defined as occurring at points in time *after* the onset of the movement (which was after the slight negative shift, cf., Fig. 7).

*Onset latencies and peaks.* As evident in Fig. 7, there are also systematic shifts of the CoP when responding verbally. These shifts are very small, but they do not merely reflect noise, as shown by the separate, symmetrical representation of the right and left responses (left part of the figure). Analysis of the onset of movement yielded a value of 236 ms for passes without a head fake and a value of 330 ms for passes with a head fake (see Fig. 7, right panel). This analysis did not reveal any initial erroneous movement for passes with head fake. However, the further course of the shifts of the CoP mirrors the course of the shifts of the CoP of the buzzer task: For passes without head fakes, a movement away from the response side with a peak at 366 ms can be observed (peak 1;—0.33 mm), followed by a shift in the direction of the response side with a peak at 752 ms (peak 2; 0.56 mm). Responding to the stimulus occurred before the second peak at 421 ms. For passes with head fakes, the pattern is identical, but there is a slight delay: Participants move away from the response side with a peak at 432 ms (peak 1; -0.29 mm), followed by a movement-shift in the direction of the response side with a peak at 814 ms (peak 2; -0.58 mm). Responding to the stimulus occurred before the second peak at 484 ms. Together, there were systematic micromovements similar to those observed in the buzzer task. However, due to the absence of erroneous shifts, these cannot be interpreted as reflecting stimulus interference in postural fluctuations. For a transparent comparison between the buzzer task and the vocal task for discrete and continuous measurements, see Fig. 8.

## Discussion

The present study was designed to investigate two specific aspects of the general framework of event coding [TEC; 6]. First, it examines which of two approaches best explains the visuo-motor process that runs through competing event files during dynamic visual input is processed: the Continuous Flow Model^7^ or Rapid-Chase Theory^8,9^. Second, the present study investigates whether the mere observation of a basketball player internally prepares the execution of an action ‘below the threshold’ or if it manifests itself in measurable (micro-)movements (i.e., slight shifts of the CoP). Of special interest was the question if even the response conflict is visible in the slight shifts of the CoP.

### Discrete measures (RT, MT, ER)

The analysis of the time measurements (RT, MT) revealed the well-known head-fake effect^4,33^, both in the buzzer task and in the vocal task. The size of the head-fake effect was similar for both experimental versions, pointing out that interference induced through the opposite head orientation is independent of the kind of response. This finding is in accordance with the study of Alhaj Alaboud et al.^36^, which showed that performing either a whole-body response-movement or ballistically pressing a response button with the index finger does not modulate the size of the head-fake effect. This finding is also in accordance with the TEC^6^, which suggests that the general nature of the spatial coding of the stimulus and response features (i.e., left/right) should not differ between whole-body response-movements and vocal responses. Consequently, the spatial stimulus codes of the pass direction and the head orientation (i.e., left, right) activated corresponding vocal-response codes (left/right) in a manner analogous to that of a whole-body response-codes, resulting in a head-fake effect in both tasks.

### Dynamic measures in the buzzer task: Evidence for early integration in a dynamic action context

The analysis of the CoP displacements in the buzzer task for passes without head fakes showed that participants shifted their CoP away from the response side before moving to the response buzzer. This initial shift is done to push off with the contralateral leg towards the response buzzer on the ipsilateral side^22^. For passes with head fakes, participants displayed a slight initial incorrect shift in the direction of the response buzzer before executing the response movement as described for passes without head fakes.

Regarding the theoretical question of whether early postural dynamics in the head-fake task resemble a strictly prime-dominated sequence^9,17^ or rather an early integration of multiple visual cues^7^, the present findings favor a cautious interpretation in terms of continuous integration. When CoP trajectories were aligned by head orientation, the early trajectories for fake and non-fake trials did not show an extended period of invariance. Instead, they diverged shortly after movement onset, and this divergence increased across successive time windows between 200 and 300 ms. In the present dynamic video context, this pattern is more compatible with early mixture or continuous integration of head orientation, body movement, and emerging pass information than with a strictly sequential prime-target account. Importantly, however, this conclusion is limited to the present dynamic sports-related paradigm. Classic Rapid-Chase Theory evidence is based on paradigms with clearly separated prime and target events and systematically varied SOAs. In contrast, the head-fake stimuli used here contain overlapping and continuously unfolding information from head rotation, trunk/body movement, and passing movement. Thus, the present study suggests that the rapid-chase independence criterion may be difficult to apply directly to naturalistic dynamic action displays, and that continuous integration may provide a more suitable description of the present head-fake situation.

This pattern is also compatible with the ecological structure of the head-fake situation. Unlike standard priming paradigms with clearly separated prime and target events, short SOAs, and discrete stimuli, the head-fake situation in basketball is a dynamic process in which head rotation, body-weight shift, and pass execution overlap in time. In such a context, it is plausible that the visuo-motor system does not rely on the processing of two strictly separate “feedforward sweeps” of visual information, but instead continuously integrates the unfolding visual information about the opponent’s action in real-time. From this perspective, the small initial misactivation observed in head-fake trials, that is, the initial shift toward the response side, followed by a rapid correction, may reflect a transient influence of the deceptive head orientation cue that is quickly attenuated as additional pass-relevant information becomes available.

### Dynamic measures in the vocal task: Observation-driven micromovements without a deceptive response conflict

In the vocal task, participants were not instructed to initiate any lateralized body movement; nevertheless, we observed systematic—albeit very small—CoP displacements with a temporal structure qualitatively similar to that observed in the buzzer task. These findings indicate that observing a dynamic lateralized action can induce measurable postural fluctuations even when no overt body movement is required. However, these micromovements should be interpreted cautiously. They are consistent with action-observation-related motor activation, including motor resonance, but they do not uniquely identify this mechanism. Alternative explanations, such as subtle attentional orienting, spatial coding of the observed action, or generalized embodied response tendencies, cannot be ruled out.

Importantly, the vocal-task CoP trajectories did not show a clear signature of deceptive response conflict, that is, an initial erroneous bias toward the head-indicated side followed by a correction. This contrasts with the buzzer task, in which participants prepared an overt lateralized movement and showed a small initial erroneous shift in head-fake trials. Thus, the present results suggest that observation alone can induce small postural dynamics, whereas a robust deceptive-bias signature in CoP emerges primarily when a lateralized action is prepared.

### Limitations

A key interpretational caveat concerns the very early divergence of the CoP trajectories between fake and non-fake trials. Despite efforts to match the videos, subtle kinematic or temporal differences between fake and non-fake sequences could provide early visual evidence that alters postural dynamics before a decisional conflict is fully established. Importantly, classic RCT evidence is based on paradigms where primes and targets do not overlap in time and where prime-target SOAs are systematically varied, revealing a prime-locked initial invariance across target characteristics (the independence criterion) across different stimulus materials^8,17^ and motor output measures^17–20^. In contrast, evidence that such rapid-chase dynamics generalize to complex whole-body movements in ecologically realistic sport contexts is still limited. Future work could address these alternative accounts more directly by parametrically manipulating the temporal relation between head- and pass-information (SOA-like manipulations within dynamic displays) and by further quantifying and controlling low-level kinematic differences between stimulus variants.

Because the present study did not include expert basketball players or non-sport control stimuli, it remains open whether the observed effects are specific to sports-related deceptive actions. Similar postural dynamics may occur with non-sport stimuli that contain conflicting directional social cues. Future work should therefore compare participants with different levels of basketball expertise and include non-sport control displays to determine whether the present effects reflect sport-specific perceptual-motor attunement or more general mechanisms of directional conflict processing.

Another limitation concerns the sample size. Although 28 participants were initially tested, 8 had to be excluded because of technical problems with force-plate recording or synchronization. These exclusions were condition-independent and unrelated to participant performance, but they reduced the final sample to 20 participants, slightly below the 22 participants, which were targeted a priori. The robust RT effects suggest that the study was sufficiently sensitive to detect the classic head-fake effect; however, smaller effects, particularly in the CoP dynamics and error rates, should be interpreted with appropriate caution.

### Conclusion

The present study extends prior head-fake research by moving beyond discrete outcome measures and examining how dynamic, sports-related deceptive cues are reflected in continuous postural adjustments. Across both tasks, we replicated the classic head-fake effect: responses were slower when head orientation and pass direction conflicted, and this was true for whole-body interceptive responses and for vocal responses alike. This cross-modality robustness supports the TEC-based interpretation that left/right stimulus features activate competing response codes irrespective of the specific effector system used to express the response. Because the participants were non-experts, the findings should be interpreted as evidence for general action-perception and response-conflict mechanisms elicited by basketball stimuli, rather than as evidence for basketball-specific expertise effects.

Overall, the combination of discrete time measures (RT/MT) and continuous CoP measurements shifts the interpretation of the head fake effect: It should not only be understood as a late interference phenomenon (longer RTs), but as a dynamic process in which early, sometimes misleading motor tendencies arise—at least in complex whole-body reactions—and are corrected online through continuous information integration. Methodologically, this underscores the added value of continuous measures in sports-related paradigms: they reveal how conflicts between event files unfold in movement, rather than merely being reflected in endpoints, as indicated by reaction times.

## Data Availability

The datasets generated during and/or analysed during the current study are available from the OSF repository: https://osf.io/v6saz/overview?view_only=cc078270409347c9bc2ad1dbf3c69298. The repository contains behavioral data (RT, MT, ER), pre-processed CoP trajectories (participant-level condition averages), analysis scripts, and diagnostic plots for onset estimation. Currently, it is a view-only link that we will make accessible after publication or upon request from the reviewers, so that files can also be downloaded.
